# The Role of Herbal Bioactive Components in Mitochondria Function and Cancer Therapy

**DOI:** 10.1155/2019/3868354

**Published:** 2019-06-12

**Authors:** Fangfang Tao, Yanrong Zhang, Zhiqian Zhang

**Affiliations:** ^1^Department of Immunology and Microbiology, Basic Medical College, Zhejiang Chinese Medical University, Hangzhou 310053, Zhejiang, China; ^2^Department of Vascular Surgery, Third Hospital of Hebei Medical University, Shijiazhuang, China; ^3^Southern University of Science and Technology, School of Medicine, Shenzhen, Guangdong 518055, China

## Abstract

Mitochondria are highly dynamic double-membrane organelles which play a well-recognized role in ATP production, calcium homeostasis, oxidation-reduction (redox) status, apoptotic cell death, and inflammation. Dysfunction of mitochondria has long been observed in a number of human diseases, including cancer. Targeting mitochondria metabolism in tumors as a cancer therapeutic strategy has attracted much attention for researchers in recent years due to the essential role of mitochondria in cancer cell growth, apoptosis, and progression. On the other hand, a series of studies have indicated that traditional medicinal herbs, including traditional Chinese medicines (TCM), exert their potential anticancer effects as an effective adjunct treatment for alleviating the systemic side effects of conventional cancer therapies, for reducing the risk of recurrence and cancer mortality and for improving the quality of patients' life. An amazing feature of these structurally diverse bioactive components is that majority of them target mitochondria to provoke cancer cell-specific death program. The aim of this review is to summarize the* in vitro* and* in vivo* studies about the role of these herbs, especially their bioactive compounds in the modulation of the disturbed mitochondrial function for cancer therapy.

## 1. Introduction

As a series of complex diseases triggered by uncontrolled cell growth and irreversible disorder of cellular homeostasis, cancer is a serious threat to human health worldwide and has become one of the leading causes of morbidity and mortality all over the world. Over the past decades, conventional therapeutic methods for cancer treatment, including surgical operation, chemotherapy, radiation therapy, hormone therapy, immunotherapy, targeted therapy, or a combination of them, have gained lots of great achievements and led to a significantly improved outcome of cancer patients to some extent. However, these therapies have numerous limitations and are not always effective due to drug resistance, cancer metastasis, and tumor relapse. On the other hand, conventional treatments often cause a variety of side effects on the healthy tissues, which indicates that new agents and novel treatment strategies are urgently needed.

In recent years, herbal drugs, including plants, herbal complexes, and biological ingredients, have drawn more attention of several scientists due to their significant therapeutic potential against various diseases, including tumors. In past few decades, medicinal herbs and their bioactive components have been successfully applied to the treatment of different types of cancer as adjuvant methods in combination with conventional therapeutic approaches, including chemotherapy, radiotherapy, targeted therapy, or immunotherapy. Many natural products extracted from these herbs have been reported to suppress cancer cell proliferation, exert beneficial effects on cancer progression, and ameliorate conventional cancer therapies-induced side effects. For instance, curcumin, a polyphenol compound derived from* Curcuma longa* is effective for the treatment of most tumors and functional on different stages of tumorigenesis, including cancer cell proliferation, invasion, and metastasis* in vitro* and* in vivo *[1]. Moreover, curcumin is highly suitable and has negligible side effects for chemoprevention of cancer.

Mitochondria are double-membrane organelles to be central to a wide range of cellular physiological processes, such as cell survival, proliferation, and migration. Mitochondrial dysfunction influences tumorigenesis, cancer cell growth, apoptosis, survival, and metastasis. As evident by the different studies done so far, altered mitochondrial function affects tumorigenesis at multiple stages. In tumor initiation stage, mutations in mitochondrial enzymes generate oncometabolites, which thus promote tumor growth and survival. For instance, Qian et al. reported that suppression of Drp1 results in cell cycle arrest at the G2/M phase due to replication stress [2]. Mitochondrial metabolic reprogramming also contributes to enhancement of the metastatic potential of tumor cells. Increased expression of Drp1 is associated with a malignant phenotype in multiple cancer types, further highlighting the role of mitochondrial dynamics in metastasis [3].

The present review highlights the application of herbal bioactive constituents as an adjuvant medicine against different types of cancer. We have focused on the regulation of mitochondrial function by these compounds, which can help in better and intensive understanding of novel approaches in chemoprevention of tumors by these ingredients.

## 2. Bioactive Compounds in Regulating the Function of Mitochondria during Tumorigenesis

### 2.1. Search Strategy

A literature search was carried out on 4/23/2019 using the Pubmed database using the following search terms: “herb & compound & mitochondrion & cancer” or “herbal & compound & mitochondrion & cancer” or “herb & compound & mitochondrion & tumor” or “herbal & compound & mitochondrion & tumor” or “herb & bioactive & mitochondrion & cancer” or “herbal & bioactive & mitochondrion & cancer” or “herb & bioactive & mitochondrion & tumor” or “herbal & bioactive & mitochondrion & tumor”. All separate searches were then combined. Titles and abstracts were read and screened using the following inclusion and exclusion criteria. A total of 46 publications were identified as directly related papers at this stage. Inclusion criteria were as follows: (1) the function of bioactive compound being reliably studied; (2) the effect of these bioactive compound being mitochondria related; (3) studies from peer reviewed journals; (4) English language. Exclusion criteria were as follows: (1) studies not being in cancer cells; (2) the molecular event of mitochondria not being mentioned; (3) bioactive compounds extracted from general food, not from herbs; (4) grey literature.

### 2.2. Homoisoflavanone-1: Natural Compound of Polygonatum odoratum

Homoisoflavanone-1 is a natural phenolic compound extracted from the Chinese medicinal herb* Polygonatum odoratum*, which has pronounced antihyperglycemic activities. Ning et al. have reported that homoisoflavanone-1 significantly induced apoptosis in A549 non-small cell lung cancer (NSCLC) cells in a dose-dependent manner. Mechanistically, treatment with homoisoflavanone-1 activated caspase-3 and decreased poly ADP-ribose polymerase, which was accompanied by a reduction in the BCL2/BAK ratio. Additionally, an increase was observed in the ER stress-related proteins PERK, ATF4, and GADD34 in A549 cells with homoisoflavanone-1 treatment, indicating that ER stress was activated in response to homoisoflavanone-1 treatment in A549 cells. Therefore, homoisoflavanone-1 induced apoptosis in A549 cells by regulating the mitochondria-caspase-dependent and ER stress pathways [4].

### 2.3. Myricetin: Flavonol Compound Found in Multiple Herbs

Myricetin (3,5,7-trihydroxy-2[3,4,5-trihydroxyphenyl]-4-chromenone) is a polyphenolic flavonoid found abundantly in plants with antioxidant, cytoprotective, antiviral, antimicrobial, and antiplatelet activities [5]. The anticancer effects of myricetin have been demonstrated against several cancer types, including leukemia, bladder cancer, liver cancer, papillary thyroid cancer, and lung adenocarcinoma [6–10]. In papillary thyroid cancer cells, treatment with myricetin induced significant cleavage of caspases 3, 8, 9, and PARP-1, suggesting that myricetin induces thyroid cancer cell death in part through the activation of caspase-dependent pathways [9]. Similar results have also been found in hepatocellular carcinoma cells [10].

### 2.4. Esculetin, Natural Coumarin Compound Found in Traditional Medicinal Herbs

Esculetin (6,7-dihydroxycoumarin), a coumarin derivative extracted from traditional medicinal herbs, has been reported to have significantly anticancer activity. In human hepatocellular carcinoma, esculetin induced apoptosis in SMMC-7721 cells by activating caspase 3 and caspase 9, promoting Bax expression, decreasing BCL-2 expression, triggering collapse of mitochondrial membrane potential, and increasing cytochrome c release from mitochondria. Moreover, the inactivation of IGF-1/PI3K/Akt signaling was observed in regulating mitochondrial dysfunction [11].

### 2.5. Curcumin: Principle Component of Curcuma longa

As an active polyphenolic compound extracted from the rhizome of turmeric, curcumin has been reported to have anticancer effects on tumorigenesis.* In vitro*, curcumin can suppress cell proliferation and induce cell apoptosis in multiple cancer cells including breast, prostate, lung, and colon cancer.* In vivo*, curcumin has been administrated into various animal models of cancer and on almost all types of organ-specific cancers. Curcumin exerts its anticancer effect through modulating numerous signaling pathways, including the estrogen receptor (ER) [12], the human epidermal growth factor receptor 2 (HER2) pathways, the apoptotic signaling cascade [13], the protein p53 (p53) signaling pathway, the nuclear factor-*κ*B (NF-*κ*B) pathway [14], the mitogen-activated protein kinase (MAPK) pathway [15], the Akt pathway [16], the Notch-1 signaling pathway [17], the Wnt/*β*-catenin signaling pathway [18], the Activator protein 1 (AP-1) signaling pathway [19], the Janus kinase/signal transducers and activators of transcription (JAK/STAT) signaling pathway [20], the Sonic Hedgehog pathway [21], the AMP-activated protein kinase/cyclooxygenase 2 (AMPK/COX-2) pathway [22], and other signaling pathways. Curcumin alone and in combination with chemotherapy or radiation therapy promote cell apoptosis in a variety of different tumors by modulating various apoptotic associated genes, including the downregulation of antiapoptosis genes Bcl-2 and the upregulation of proapoptosis genes PUMA, Bax, and caspase cascades. For instance, curcumin suppresses laryngeal cancer cell proliferation and induces apoptosis through downregulating Bcl-2 and PI3K/Akt [23]. In breast cancer, curcumin enhances mitomycin C sensitivity in breast cancer stem cells also by inducing Bcl-2 mediated apoptosis [24]. Moreover, curcumin can also activate p53 to regulate cell proliferation, autophagy, and apoptosis in gastric cancer cells. Furthermore, curcumin can directly react with reactive oxygen species (ROS) to increase the expression of apoptosis receptors on the cancer cell membrane [25].

To determine which apoptosis pathway was triggered by curcumin, Gogada et al. treated human MDA-MB231, PC3, and LNCaP cells with 15 *μ*M of curcumin and found that curcumin increased the expression of apoptosis protease-activating factor-1 (Apaf-1), the activity of caspase 9, and the release of cytochrome c. However, caspase 8 knockout cells showed no effect in curcumin-induced caspase activation and cell death activation, suggesting that curcumin modulates the apoptosis through the mitochondria-dependent pathway [26].

### 2.6. [6]-Gingerol: Bioactive Component of Ginger

[6]-Gingerol is a pharmacologically impressive bioactive component derived from ginger, which have been shown to have antihyperglycemic, antioxidative, and anticancer properties. Chakraborty et al. found that treatment of metastatic cervical cancer HeLa cells with [6]-gingerol induced several morphological changes, including externalization of phosphatidylserine to the cell surface, DNA fragmentation and significant increase of TUNEL positive cells, depolarization of mitochondrial membrane potential, and increased expression of caspase 3 and PARP [27]. Moreover, in mouse skin tumorigenesis model, [6]-gingerol treatment [6]-gingerol possesses apoptotic potential by releasing of cytochrome c, activating caspases, increasing apoptotic protease-activating factor-1 (Apaf-1) [28]. Studies from Ma laboratory showed that mitochondria and lysosome may be the primary targets for [6]-gingerol in hepatoma G2 (HepG2) cells. In addition, cathepsin D played a crucial role in the process of [6]-gingerol induced apoptosis in HepG2 cells. The release of cathepsin D to the cytosol appeared to be a upstream of cytochrome c release from mitochondria [29].

### 2.7. Triptolide: Natural Product of Tripterygium wilfordii Hook f.

Triptolide is a diterpenoid triperoxide extracted from* Tripterygium wilfordii Hook f.* and exerts broad-spectrum anticancer activities. In nasopharyngeal carcinoma, triptolide induced cell cycle arrest at S phase, and promoted cell apoptosis via the caspase 9 dependent pathway [30]. Kong et al. found that triptolide decreased the mitochondrial membrane potential and induced Bax translocation to mitochondria via the SIRT3-GSK-3*β* cascade [31]. Moreover, triptolide treatment resulted in cell cycle arrest at G2/M phase and mitochondrial membrane depolarization in murine pituitary corticotroph tumor cells through the NF-*κ*B signaling pathway [32]. In non-small cell lung cancer cells, triptolide decreased mitochondrial respiration by modulating SIRT3 in a p53-dependent manner [33]. In p53-deficient cells, triptolide impaired the translocation of SIRT3 from cytosol to mitochondria and decreased the enzymatic activities of complexes I and II components by acetylation [33]. Chan et al. demonstrated that triptolide was cytotoxic through inducing cell cycle arrest at G0/G1 phase and led to cell autophagy and apoptosis by causing the release of Ca^2+^, the production of ROS, and the depolarization of mitochondrial membrane* in vitro* in murine leukemia WEHI-3 cells [34]. In liver, the metabolism of triptolide is mediated by cytochrome P450. Interestingly, due to different isoforms of P450 in males and females, female animals are more sensitive to triptolide [35, 36].

### 2.8. Thymoquinone: Active Constituent of Nigella sativa Seeds

Thymoquinone, an active ingredient derived from the seeds of* Nigella sativa*, has been reported to show antimicrobial, antioxidant, antitumor, and anti-inflammatory activities and have been widely utilized in dealing with diverse diseases. Thymoquinone exhibits cytotoxic effects by inhibiting cell proliferation and inducing cell apoptosis in several cancer types, including bladder cancer [37], colon cancer [38], pancreatic cancer [39], neuroblastoma [40], osteosarcoma [41], myeloblastic leukemia [42], and acute lymphoblastic leukemia [43]. In T24 and 253J bladder cancer cells, thymoquinone changed the protein levels of Bax, Bcl-2, and cytochrome c and resulted in mitochondrial dysfunction. Pretreatment with Z-VAD-fmk, a pan-caspase inhibitor, could partially reverse the apoptosis-promoting effect of thymoquinone by inducing the expression of antiapoptotic protein Bcl-2, suppressing the translocation of Bax from the cytoplasm to mitochondria and blocking the release of cytochrome c [37]. Thymoquinone was also found to trigger mitochondrial outer membrane permeability and activate autophagic cell death in irinotecan-resistant (CPT-11-R) LoVo colon cancer cell line [38]. Pretreatment of thymoquinone and betulinic acid along with gemcitabine synergistically suppressed the cancer cell proliferation* in vitro* by downregulation of PKM2 expression, a promising component of cellular metabolism [39]. In acute lymphocyte leukemic CEM-ss cells, thymoquinone triggered mitochondrial apoptosis through the production of cellular ROS and activation of caspases 3 and 8 [43]. Activated caspase 8 also initiated cytochrome c release during thymoquinone-induced apoptosis in p53-null HL-60 cancer cells [42].

### 2.9. Epigallocatechin Gallate: Representative Polyphenol of Green Tea

Green tea is the most widely used beverage worldwide, which is reported to play an effective role in the prevention of tumorigenesis in many types of carcinomas [44]. Epigallocatechin gallate (EGCG), an active ingredient of green tea, has attracted much attention of researchers for its abilities in antiproliferation, antimetastasis, and proapoptosis in multiple diseases including cancer [45]. As an indicator of hepatocellular carcinoma (HCC), high levels of *α*-fetal protein (AFP) are related to malignant differentiation and poor prognosis of cancer cells [46]. Wang et al. demonstrated that EGCG could effectively stimulate autophagy by reducing *α*-fetal protein (AFP) secretion and simultaneously inducing degradation of AFP aggregation in human HCC HepG2 cells [46]. In B lymphoma cells, EGCG also could induce cell apoptosis via mitochondrial and death receptor pathways [47]. Importantly, pretreatment with EGCG obviously maintained normal mitochondrial function in male Wistar rats [48]. Interestingly, in human laryngeal epidermoid carcinoma Hep-2 cells, EGCG did not activate caspase, nor did change the intracellular ROS level. Treatment of EGCG of Hep-2 cells elevated the expression level of p53, with a decrease protein levels of Bcl-2 and Bid and an increase level of Bax. Moreover, EGCG treatment induced cytochrome c from the mitochondria to cytosol with a decreased mitochondrial membrane potential and, subsequently, increased translocation of apoptosis-inducing factor (AIF) and endonuclease G (EndoG) into the nucleus to provoke cellular apoptosis. These data suggest that EGCG-induced apoptosis of human laryngeal epidermoid carcinoma Hep-2 cells is caspase-independent and the p53-mediated mitochondrial pathway and the nuclear translocation of AIF and EndoG are critical [49].

### 2.10. Artepillin C: Bioactive Component of Brazilian Green Propolis

Artepillin C is a bioactive component of Brazilian green propolis which possesses antitumor and chemopreventive activities [50]. In prostate cancer cells, artepillin C sensitized the TRAIL-resistant LNCaP cells by engaging both the intrinsic (mitochondrial) apoptotic pathway and the extrinsic (receptor-mediated) pathway [51]. Artepillin C enhanced the expression of TRAIL-R2, reduced the activity of NF-*κ*B, induced the significant activation of caspases 3 and 8, and disrupted ∆*ψ*m of mitochondria [51]. In HepG2 cells, artepillin C prevented oxidative damage and suppressed lipid peroxidation in a dose-dependent manner [51]. Interestingly, artepillin C exerts its antileukemic effects partially via enhancing the expression of Fas antigen and disrupting the mitochondrial membrane potential [52].

### 2.11. Allicin: Predominant Component of Freshly Crushed Garlic

Allicin is one of the most biologically active compounds of freshly crushed garlic [53]. In hepatocellular carcinoma (HCC), it has been reported that allicin has protective effect and antitumor activity. Combined treatment of HCC cells with allicin and 5-fluorouracil (5-FU) increased intracellular ROS level, activated caspase 3, and PARP reduced Bcl-2 and decreased the mitochondrial membrane potential, compared with DMSO, 5-FU, and allicin treated alone, which suggest that allicin enhanced HCC cells to 5-FU induced apoptosis via ROS-mediated mitochondrial pathway [54]. Allicin also induced p53-guided autophagy in HCC cell lines [55]. Western blotting results indicated that allicin decreased the level of cytoplasmic p53 and Bcl-2, blocked the PI3K/mTOR signaling pathway, and increased the expression of AMPK and Beclin-1 signaling pathways in HepG2 cells, which results in allicin-induced mitochondria degradation [55]. In human neuroblastoma SK-N-SH cells, allicin-induced cell apoptosis through phosphorylation of p38 MAPK, activating caspases 3 and 9 and subsequently inducing the release of cytochrome c from mitochondria into the cytosol [56]. Moreover, experiment results have demonstrated that allicin was chemopreventive to gastric cancer by suppressing cancer cell growth, arresting cell cycle at G2/M phase, inducing endoplasmic reticulum (ER) stress, scavenging free radicals and promoting mitochondria-mediated apoptosis [57]. Xu et al. demonstrated that allicin guided SKOV3 cell apoptosis through the phosphorylation of JNK in a time- and dose-dependent manner. Phosphorylated JNK activated Bcl-2 family, led to the mitochondrial translocation of Bax and cytochrome c release and subsequently triggered mitochondria-mediated signaling pathways [58]. In colon cancer HCT-116 cells, treatment with allicin enhanced hypodiploid DNA content, downregulated Bcl-2, upregulated Bax, increased cytochrome c release from mitochondria to the cytosol, and induced translocation of NF-E2-related factor-2 (Nrf2) to the nuclei, which result in apoptotic cell death [59].

### 2.12. Ganoderma atrum Polysaccharide: Active Ingredient of Ganoderma atrum

*Ganoderma atrum* polysaccharide (PSG-1), the major bioactive component of* Ganoderma atrum*, has attracted great attention for its pronounced antitumor activity [60]. Zhang et al. showed that treatment of colon adenocarcinoma CT26 cells* in vitro* with PSG-1 exhibited no effect on cell proliferation directly. However, administration of PSG-1 significantly suppressed xenograft tumor growth through induction of apoptosis in CT26 tumor-bearing mice* in vivo*. The authors found that PSG-1 induced apoptosis was associated with elevation of p53 and Bax expression, downregulation of Bcl-2, activation of caspases 3 and 9, mitochondrial membrane potential loss, mitochondrial cytochrome c release, and intracellular ROS production. In addition, PSG-1 increased immune organ index, induced lymphocyte proliferation, and enhanced cytokine levels in serum. These data suggest that PSG-1 exerts its potential antitumor activity* in vivo* by inducing mitochondria-mediated apoptosis and enhancing systematic immune functions [61–63]. Similar results were also observed in sarcoma 180-bearing mice [64].

### 2.13. Ginsenosides: Major Pharmacologically Active Ingredients of Ginseng

As a herbal drug of TCM, ginseng has long been used extensively to support human energy [65]. Ginseng has been associated with a decreased incidence of cancers, diabetes, cardiovascular diseases, and neurodegenerative disorders [66]. Ginsenosides, the major pharmacologically active ingredients of ginseng, have been shown to have anti-inflammation, antioxidation, and anticarcinogenic activities [66]. In the non-small cell lung cancer (NSCLC) cells and H460 xenograft tumor model, the apoptosis-inducing effects of ginsenoside Rk3 were triggered by mitochondria-dependent pathways, in which Rk3 decreased Bcl-2 expression, increased Bax expression, caused cytochrome c release, induced caspases 3, 8, and 9 activation, and promoted mitochondrial membrane potential changes [67]. Moreover, ginsenosides also induced mitochondrial-associated apoptosis by increasing mitochondrial ROS in human leukemia [68], prostate [69], colorectal [70], and neuroblastoma [71] cancer cells.

### 2.14. Jolkinolide B, Diterpenoid from Euphorbia fischeriana Steud.

Jolkinolide B (JB), a bioactive compound extracted from the roots of* Euphorbia fischeriana Steud*, has been found to inhibit tumor growth via multiple pathways [72–77]. In colorectal carcinoma (CRC) cells, JB suppressed the cell viability and colony formation of CRC cells, as determined by Annexin V/PI and confirmed by increased expression of cleaved caspase 3 and cleaved-PARP. Mechanistically, JB provoked ROS generation, and suppression of ROS generation with N-acetyl L-cysteine could reverse the JB-induced apoptosis. Moreover, JB treatment increased intracellular and mitochondrial Ca^2+^ level and decreased the mitochondrial membrane potential in CRC cells [74]. JB also induced tumor cell apoptosis, downregulated the expression of glucose transporter genes (Glut1, Glut3, and Glut4), glycolysis-related kinase genes (Hk2 and Ldh-a) and antiapoptosis genes (Bcl-2, caspases 3 and 9), upregulated the mRNA expression of proapoptosis genes (Bax) and ROS production, and decreased the potential of mitochondrial membrane, ATP, and lactic acid production in mouse melanoma B16F10 cells [72].

### 2.15. Withaferin A Steroidal Lactone of Withania somnifera

Withaferin A (WA), an active steroidal lactone derived from the herbal plant* Withania somnifera*, possesses antitumorigenic, immunomodulatory, and reactive oxygen species (ROS) modulating activities against various cancer cells [78]. In breast cancer cell, WA treatment suppressed polyethylene glycol- (PEG-) induced mitochondrial fusion and Complex III assembly accompanied by reducing the expression of mitofusin1 (Mfn1), mitofusin2 (Mfn2), and optic atrophy protein 1 (OPA1), which are involved in fusion process of mitochondria [79]. Xia et al. also demonstrated that WA directly suppressed cell growth and induced cell apoptosis in colorectal cancer cells by disrupting the ROS-mediated mitochondrial function and JNKs signaling pathway [80]. In skin epidermal JB6 P+ cells, a well-established model for tumor promotion, WA inhibited tumor promoter TPA-induced decreases in IDH1 activity and mitochondrial function [81]. In breast cancer MCF-7 and MDA-MB-231 cells, WA-induced cell death by ROS-mediated paraptosis [82], which is characterized by dilation of endoplasmic reticulum and mitochondria, followed by fusion and formation of larger vacuoles and lack of autophagic or apoptotic morphology [82]. WA-induced paraptosis may afford an effective therapeutic approach for the treatment of breast cancer. In prostate cancer, WA could kill metastatic castration-resistant cancer cells but not normal cells through increased expression level of c-Fos and the production of ROS and decreased FLIP level [83]. In human melanoma cells, Mayola et al. showed that the apoptotic process triggered by WA included the mitochondrial-mediated apoptosis pathway associated with DNA fragmentation, Bcl-2 downregulation, caspase 9 and caspase 3 activation, Bax translocation, cytochrome c release, and disruption of mitochondrial transmembrane potential [84].

### 2.16. Cucurbitacin B: A Bioactive Compound from Pedicellus Melo

Cucurbitacin B (CuB) is a naturally bioactive compound that is found abundantly in cucumbers and the traditional Chinese herbal medicine,* Pedicellus Melo*, and it is known to exhibit antitumor and anti-inflammation effects in several human cancers as a small molecule of STAT3 inhibitor [85]. In prostate cancer, CuB significantly and specifically inhibited prostate cancer cell growth by inhibiting ATP citrate lyase (ACLY) phosphorylation. Ectopic expression of ACLY abrogated CuB's apoptotic effects in prostate cancer cells [86]. In addition, CuB induced HUVEC apoptosis and may induce apoptosis by triggering the mitochondrial apoptotic pathway [87].

## 3. Mechanistic Insights on Mitochondrial Induced Cell Death Processes by These Natural Compounds

### 3.1. Regulation of Mitochondrial Energy Metabolism

Mitochondria are the main organelles to produce ATP, which provide 95% of the energy for cell activities [88]. Tumor cells are characterized by uncontrolled growth, which is facilitated by dysfunctional mitochondria in two different manners: apoptosis surveillance and metabolism chaos. Mitochondrial energy metabolism mainly includes the tricarboxylic acid cycle oxidative respiratory chain and oxidative respiratory chain. Therefore, the regulation of mitochondrial energy metabolism is primarily achieved by regulating the activity of critical enzymes in different energy metabolism pathways. The enzymes involved in mitochondrial energy metabolism mainly include lactate dehydrogenase (LDH), succinate dehydrogenase (SDH), ATPase, and ATP synthase. Gossypol is a polyphenolic compound extracted from cotton seeds and is a nonselective competitive LDH-A inhibitor with no significant toxic effect on normal tissues and its antitumor activity seems to be associated with LDH-A inhibition [89]. Multiple bioactive compounds modulate the activity of LDH. For instance, ginsenoside Rb1 could reduce LDH release and block the effect of LDH activity, showing protective effects on human umbilical vein endothelial cells* in vitro *[90]. Baicalin significantly reduces the infarct size and LDH in rats with myocardial infarction of rats [91]. ATPases also play an important role in processes such as catalytic energy conversion, material transport, and information transfer. Pathological states such as hypoxia and disease suppress mitochondrial energy metabolism and ATPase activity is correspondingly reduced. Rb1 can facilitate ATP production in adipose cells [92]. Prenylflavonoid suppressed inhibited ABCB1-mediated doxorubicin efflux and stimulated the ATPase activity of ABCB1 in breast MCF-7/ADR cells [93].

Tumor cell metabolism has been considered a hallmark of cancer to promote cancer growth and survival. Increased glucose uptake and fermentation of glucose to lactate are the two common features of cancer cells, which is known as “Warburg Effect”, that means cancer cells could predominantly produce energy by glycolysis even in the presence of oxygen [94]. In mouse ovarian surface epithelial (MOSE) cancer, sphingosine, a bioactive sphingolipid metabolite, was found to decrease the citrate synthase activity, cholesterol synthesis, and glycolysis and increased TCA flux [95]. In ovarian cancer cells, 20(S)-Rg3 upregulates miR-532-3p via suppressing DNMT3A-mediated DNA methylation and thus promoted the direct inhibition of miR-532-3p on HK2 to antagonize the Warburg effect [96]. In prostate cancer cells, SK1, one isoform of the sphingosine kinase, functions to maintain the Warburg effect and cell survival via increasing the stability of c-Myc and suppressing Ap3A formation [97]. HIF-1 is overexpressed in various types of cancer and the levels of its activity have already been demonstrated closely to glycolytic activity. Apigenin, a plant flavonoid compound, is considered as a typical HIF-1*α* inhibitor in human prostate and ovarian cancers [98]. EGCG could suppress the expression of HIF-1*α* by blocking the interaction between Hsp90 and HIF-1*α* [99]. Several studies indicate that curcumin inhibited the mRNA level of HIF-1*α* and HIF-1*β* genes [100, 101].

### 3.2. Regulation of Oxidative Stress

Oxidative stress (OS) is induced an imbalance between the generation and elimination of reactive oxygen species (ROS). Excessive production of ROS may result in DNA, protein, and lipid damage and genetic alterations that can favor initiation, promotion, and progression [102, 103]. A number of phytochemicals have been shown to exert antioxidant properties via modulating ROS-mediated signaling pathways during all phases of tumorigenesis. Curcumin simultaneously reduces the level of OS in cancer cells by regulating multiple downstream pathways. For instance, in pancreatic cancer cell PANC1 and BxPC3, curcumin suppresses ROS-mediated ERK and NF-*κ*B activation and their downstream genes MMP2 and MMP9 and thus inhibits cell invasion and migration [104]. Curcumin suppressed cancer-associated fibroblasts- (CAFs-) induced prostate cancer cell epithelial-to-mesenchymal transition by inhibition of ROS-induced MAOA/mTOR/HIF1a signaling [105]. In liver carcinoma HepG2 cells, EGCG has been shown to reduce H2O2-mediated cytotoxicity via increasing cellular GSH level [106]. Moreover, EGCG suppresses colon cancer cell growth and metastasis by activating Nrf2-UGT1A signaling [107].

### 3.3. Regulation of Apoptosis in the Mitochondrial Pathway

There are two main pathways involved in cell apoptosis: cell death receptor pathway and mitochondrial pathway. The mitochondria-mediated apoptosis pathway acts as the main switch of apoptosis is mainly managed by release of cytochrome C and disruption of the mitochondrial membrane potential [108]. Moreover, antiapoptotic protein Bcl-2 and its family members such as Bax, Bid, Bak, Bad, Bim, and Bmf are very important players. In bladder cancer cells, cisplatin and curcumin significantly increased apoptosis by caspase-3 activation, increased protein levels of p53 and p21, and decreased protein levels of pSTAT3 [109]. Lariciresinol (the main active ingredients in* Patrinia*) is a promising anticancer drug in HCC targeting to the mitochondrial-mediated apoptosis pathway [108].

### 3.4. Regulation of Mitochondrial Membrane Permeability Transition Pores (MPTP)

Growing evidence demonstrates that the mitochondrial permeability transition pore (PTP) is involved in the initiation and progression of tumors. The MPTP is a highly conductive giant channel composed of the voltage-dependent anion channel (VDAC), the adenine nucleotide translocator (ANT), and cyclophilin D. The opening of MPTP is usually induced by ROS and Ca^2+^ overload. These changes induce mitochondrial apoptosis and ROS release [110–112]. Icaritin (ICT), a common aglycone isolated from the Chinese plant* Epimedium*, potently inhibited proliferation and survival of colorectal cancer cells. Zhou et al. show that ICT treatment in CRC cells induces MPTP opening and subsequent CRC cell necrosis [112].

## 4. Conclusion and Future Perspective

**Figure 1:**
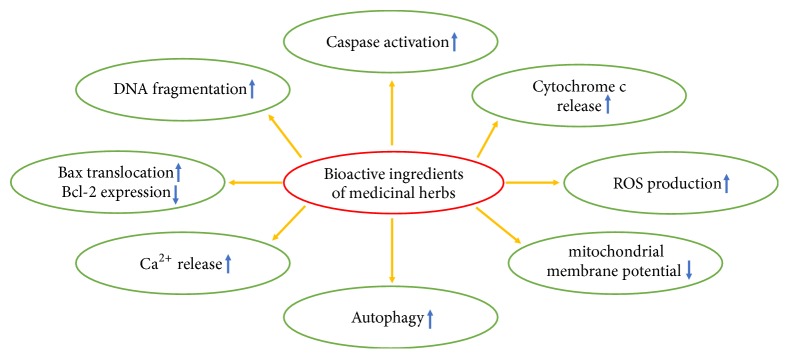
Mitochondria associated mechanisms of herbal bioactive components under cancer prevention.

In this study, we summarized the role of bioactive components from natural plants in cancer prevention and treatment, especially the effects and mechanisms of these compounds on mitochondrial morphology and functions (Table 1 and Figure 1). Substantial evidence demonstrates that bioactive compounds from natural plants have been used successfully to prevent and treat cancer. These natural sources are not only the potentially rich and important sources for drug screening but also beneficial for further understanding the molecular mechanisms underlying related cancers. Importantly, understanding the molecular events involved in bioactive components eventually allow developing innovative antitumor remedies and personalized interventions against cancers. Indeed, the effects of natural plants and their bioactive components on human health and disease cure are rather complex as in many other western drugs. However, with the development in science and technology, novel techniques such as high throughput screening have been applied to extract and isolate the bioactive compounds from natural sources. In combination with molecular methods and technologies, we can elucidate the underlying mechanisms more clearly to design and develop an accurate targeted therapeutic agent and reduce the side-effect of these agents.

## Figures and Tables

**Table 1:** Bioactive components of natural plants in cancer treatment through modulation of mitochondrial functions.

Bioactive components	Medicinal herbs	Effect/Mechanism/Targets associated with mitochondria	Treated cancers

Homoisoflavanone-1	*Polygonatum odoratum*	Activated caspase-3, Poly ADP-ribose polymerase, BCL2, BAK, ER, PERK, ATF4, and GADD34	Non-small cell lung cancer

Myricetin	Multiple herbs	Caspases 3, 8, 9, and PARP-1	leukemia, bladder cancer, liver cancer, papillary thyroid cancer and lung adenocarcinoma

Esculetin	Multiple herbs	Active caspase-3, active caspase-9, BAX, BCL-2, Collapse of mitochondrial membrane potential, Cytochrome c, IGF-1/PI3K/Akt	Hepatocellular carcinoma

Curcumin	*Curcuma longa*	ER, HER2, p53, NF-*κ*B, MAPK, Akt, Notch-1, Nrf2, Wnt/*β*-catenin, AP-1, JAK/STAT, Sonic Hedgehog, AMPK, Bcl-2, PUMA, Bax, ROS, Apaf-1, Cytochrome c	Breast, prostate, lung, colon

[6]-Gingerol	*Ginger*	DNA fragmentation, mitochondrial membrane potential, Caspase-3, PARP, Cytochrome c, Apaf-1,	Cervix, skin, liver,

Triptolide	*Tripterygium wilfordii Hook f.*	Cell cycle arrest, Caspase-9, Bax, SIRT3-GSK-3*β*, mitochondrial membrane depolarization, NF-*κ*B, p53, Ca^2+^, ROS, P450	Nasopharynx, murine pituitary corticotroph, lung, leukemia,

Thymoquinone	*Nigella sativa seeds*	Bax, Bcl-2, Cytochrome c, mitochondrial outer membrane permeability, autophagy, PKM2, ROS, Caspase-3, Caspase-8	Bladder, colon, pancreas, neuroblastoma, osteosarcoma, myeloblastic leukemia, acute lymphoblastic leukemia

Epigallocatechingallate	*green tea*	AFP, autophagy,	Liver, B lymphoma

Artepillin C	*Brazilian green propolis*	TRAIL, TRAIL-R2, NF-*κ*B, Caspase-3, Caspase-8, ∆*ψ*m of mitochondria, oxidative damage, lipid peroxidation, Fas antigen	Prostate, liver, leukemia

Allicin	*freshly crushed garlic*	ROS, Caspase-3, Caspase-9, PARP, Bcl-2, mitochondrial membrane potential, p53-guided autophagy, PI3K/mTOR, AMPK, Beclin-1, p38 MAPK, Cytochrome c, endoplasmic reticulum (ER) stress, free radicals, JNK, Bax, DNA content, Nrf2	Liver, neuroblastoma, stomach, ovary, colon

Ganoderma atrum polysaccharide	*Ganoderma atrum*	p53, Bax, Bcl-2, Caspase-3 and Caspase-9, mitochondrial membrane potential, Cytochrome c, ROS	Colon, sarcoma

Ginsenosides	*Ginseng*	Bcl-2, Bax, Cytochrome c, Caspase-3, Caspase-8, and Caspase-9, mitochondrial membrane potential, ROS	Lung, leukemia, prostate, colon, neuroblastoma

Jolkinolide B	*Euphorbia fischeriana Steud*	Caspase-3, PARP, ROS, Ca^2+^, Glut1, Glut3, Glut4, Hk2, Ldha, Bcl-2, Caspase-9, Bax, ATP, lactic acid production, mitochondrial membrane potential	Colon, melanoma

Withaferin A	*Withania somnifera*	ROS, mitochondrial fusion, Complex III assembly, Mfn1, Mfn2, OPA1, JNK, IDH1, paraptosis, c-Fos, FLIP, DNA fragmentation, Bax	Breast, Colon, Skin

Cucurbitacin B	*Pedicellus Melo*	ACLY, STAT3	Prostate cancer
